# Off-Centered Stagnation Point Flow of a Couple Stress Fluid towards a Rotating Disk

**DOI:** 10.1155/2014/163586

**Published:** 2014-02-03

**Authors:** Najeeb Alam Khan, Fatima Riaz

**Affiliations:** Department of Mathematical Sciences, University of Karachi, Karachi 75270, Pakistan

## Abstract

An investigation has been made to study the off-centered stagnation flow of a couple stress fluid over a rotating disk. The model developed for the governing problem in the form of partial differential equations has been converted to ordinary differential equations with the use of suitable similarity transformation. The analytical approximation has been made with the most promising analytical approach, homotopy analysis method (HAM). The convergence region of the obtained solution is determined and plotted. The effects of couple stress and nondimensional parameters have been observed on the flows of couple stress fluid. Also comparison has been made with the Newtonian fluid as the special case of considered problem.

## 1. Introduction 

The hydrodynamic flow of a viscous fluid due to the rotation of an infinite disk was the subject initially considered by Von Kármán [1] and has extensive applications in the fields of technology, engineering, industries, aerodynamics, and so forth. The work of Von Kármán has been extended to the immense directions by considering various forms of fluids, as well as diverse disk types [2–5]. The need of the century is to consider and investigate the non-Newtonian couple stress fluids which have wide real life applications in flow problems. The researchers are now paying adequate attention in this dimension and adding pioneer works in the literature in this regard. Stokes [6] was the first who developed the couple stress fluid theory which preserves couple stresses and body couples and represents the simplest generalization of the classical fluid theory. Extrusions of polymer fluids, cooling of metallic plates in a bath, rotating machinery, and so forth are the applications of couple stress fluids. Sajid et al. [7] provided the study on some two-dimensional flows with couple stresses; Malashetty et al. [8] investigated the double diffusive convection in a couple stress fluid saturated porous layer with Soret effect; melting heat transfer in the boundary layer flow of a couple stress fluid over a stretching surface is considered by Hayat et al. [9]; Khan and colleagues found the approximate solution of the couple stress fluid between expanding and contracting walls [10]; an analysis has been provided for three-dimensional magnetohydrodynamics flow of couple stress fluid with Newtonian heating by Ramzan et al. [11].

Blunt rotating bodies moving in a fluid produce the forced flow over rotating bodies in which stagnation region experiences the highest pressure, high heat transfer, and so forth, making it significant to consider it as a research problem. Hannah [12] was the first to examine the forced flow and explain the axisymmetric stagnation point flow towards a rotating disk. Wang [13] investigated the nonaligned stagnation point flow towards a rotating disk and found that the flow configuration became complicated by nonalignment. Dinarvand [14] reevaluated the Wang [13] results by employing the homotopy analytical approach.

Homotopy analytical approach presented by Liao [15] is the most effective technique to solve nonlinear equations as it provides wide flexibility to choose the convergence region of the obtained series solution with the help of *ħ*, “the convergence control parameter.” The technique is a powerful tool to acquire the analytical solution of the problem which converges relatively close to the exact solutions. Many investigations have been made [16] to scrutinize its convergence and found it relatively convenient as compared to other techniques.

In present study, the couple stress fluid has been investigated for the stagnation flow towards the off-centered rotating disk. The current investigation is an extension in the previous work of Wang [13] with the replacement of viscous fluid to couple stress fluid. The three-dimensional partial differential equations with body stresses and body couples are transformed to ordinary differential equations with the use of similarity transformation. The homotopy analysis method is employed to obtain the analytic solution of the governing problem in the form of series. The convergence analysis has also been done to determine the convergence region of the achieved solution. The influences of pertinent parameters on the velocity profiles are examined through graphs. The considered problem will recover the problem of Wang [13] as a special case.

## 2. Problem Formulation

**Figure 1:**
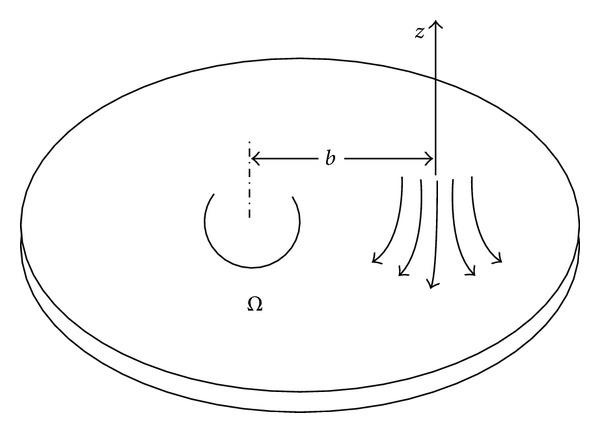
Off-centered stagnation flow on a rotating disk.

Consider the steady, incompressible, and off-centered stagnation point flow of a couple stress fluid over an infinite disk rotating with angular velocity *Ω*. The stagnation flow is at distance *b* along *z*-axis intruding on a rotating disk (shown in Figure 1). Let *u*, *v*, and *w* be the velocity components along *x*, *y* and *z* directions, respectively. The partial differential equation governing the problem can be described as

(1) $$\begin{array}{c} \nabla \cdot V=0 \end{array}$$

(2) $$\begin{array}{c} \rho \left(\frac{\partial V}{\partial t}+\left(V\cdot \nabla \right)V\right)=-\nabla p+\mu \nabla^{2}V-\gamma \nabla^{4}V, \end{array}$$

where ∇^2^ = (∂^2^/∂*x* ^2^)+(∂^2^/∂*y* ^2^)+(∂^2^/∂*z* ^2^) and ∇^4^ = (∂^4^/∂*x* ^4^)+(∂^4^/∂*y* ^4^)+(∂^4^/∂*z* ^4^) + 2(∂^4^/∂*x* ^2^∂*y* ^2^) + 2(∂^4^/∂*y* ^2^∂*z* ^2^) + 2(∂^4^/∂*x* ^2^∂*z* ^2^) subject to the boundary conditions near the disk are

(3) $$\begin{array}{c} u=-\Omega y,\;\;v=\Omega \left(x-b\right),\;\;w=0, \end{array}$$

and at infinity

(4) $$\begin{array}{c} u=ax,\;\;v=ay,\;\;w=-2az, \end{array}$$

where *a* represents the strength of the stagnation flow.

The similarity transformations presented by Wang [13] are

(5) $$\begin{array}{c} u=axf^{\prime }\left(\eta \right)-\Omega yg\left(\eta \right)+b\Omega k\left(\eta \right),\\ v=ayf^{\prime }\left(\eta \right)+\Omega xg\left(\eta \right)+b\Omega h\left(\eta \right),\\ w=-2\sqrt{a\nu }f\left(\eta \right), \end{array}$$

where *ν* is the kinematic viscosity and

(6) $$\begin{array}{c} \eta =z\sqrt{\frac{a}{\nu }}. \end{array}$$

Using the above similarity transformations in (1), the equation of continuity is satisfied whereas the governing partial differential equation reduced to the following equations:

(7) $$\begin{array}{c} f^{\prime \prime \prime }-f^{\prime 2}+\alpha^{2}g^{2}+2ff^{\prime }-\lambda f^{\prime \prime \prime \prime \prime }+1=0,\\ g^{\prime \prime }-2gf^{\prime }+2fg^{\prime }-\lambda g^{\prime \prime \prime \prime }=0,\\ k^{\prime \prime }-kf^{\prime }+\alpha hg+2fk^{\prime }-\lambda k^{\prime \prime \prime \prime }=0,\\ h^{\prime \prime }-\alpha gk-hf^{\prime }+2fh^{\prime }-\lambda h^{\prime \prime \prime \prime }=0, \end{array}$$

where *α* = *Ω*/*a* is nondimensional rotational parameter and *λ* = *aγ*/*ν* ^2^ *ρ* is the non-dimensional couple stress parameter. The boundary conditions take the form

(8) $$\begin{array}{c} f\left(0\right)=0,\;\;f^{\prime }\left(0\right)=0,\;\;f^{\prime }\left(\infty \right)=1,\\ g\left(0\right)=1,\;\;g\left(\infty \right)=0,\\ k\left(0\right)=0,\;\;k\left(\infty \right)=0,\\ h\left(0\right)=1,\;\;h\left(\infty \right)=0, \end{array}$$

where primes denote the differentiation with respect to *η*.

Equations (7) represent the system of couple stress equations. However, if the value of couple stress parameter *λ* is set to zero in the presented system, (7) will be reduced to the Navier-Stokes equations of stagnation flow [13].

The pressure *p* can be recovered by

(9) $$\begin{array}{c} p=p_{0}-\frac{1}{2}\rho a^{2}\left(x^{2}+y^{2}\right)-\frac{1}{2}\rho \left(w^{2}-2\nu w_{z}+2\gamma^{\prime }w_{zzz}\right), \end{array}$$

where *p*_0_ is the pressure at the origin and *ρ* is the fluid density. The shear stress on the disk for the couple stress fluid is given by

(10) τx=(ρν∂u∂z−γ∂3u∂z3)|z=0=ρaνa[xf′′(0)−αyg′(0)+bαk′(0)]+a2aγν3/2[−xf′′′′(0)+αyg′′′(0)−bαk′′′(0)],τy=(ρν∂v∂z−γ∂3v∂z3)|z=0=ρaνa[yf′′(0)+αxg′(0)+bαh′(0)]−a2aγν3/2[yf′′′′(0)+αxg′′′(0)+bαk′′′(0)].

The shear centre can be obtained by setting (10) to zero and solving for (*x*, *y*) as the shear stress is zero at the centre. The torque experienced by the disk of radius *R* is

(11) $$\begin{array}{c} M=\int_{0}^{R}\int_{0}^{2\pi }\left(\tau_{y}\cos\theta -\tau_{x}\sin\theta \right)r^{2}d\theta \;dr, \end{array}$$

where (*r*, *θ*) are cylindrical coordinates. Since *x* = *r*cos⁡*θ* − *b* and *y* = *r*sin*θ*, we find the torque as

(12) $$\begin{array}{c} M=\frac{\pi }{2}\frac{a\sqrt{a}}{\nu^{3/2}}\alpha R^{4}\left(\nu^{2}\rho g^{\prime }\left(0\right)-a\gamma g^{\prime \prime \prime }\left(0\right)\right), \end{array}$$

which is unaltered by the non-aligned disk axis and flow axis.

## 3. Analytical Approximations by means of HAM

The auxiliary linear operators, *ℒ*_1_[*f*] and *ℒ*_2_[*g*, *k*, *h*], are selected for (7):

(13) $$\begin{array}{c} {ℒ}_{1}\left[f\right]=\frac{d^{3}}{d\eta^{3}}-\frac{d}{d\eta },\;\;{ℒ}_{2}\left[g,h,k\right]=\frac{d^{2}}{d\eta^{2}}-1, \end{array}$$

and also the initial guesses satisfying the initial conditions in (8) are

(14) $$\begin{array}{c} f_{0}\left(\eta \right)=\eta -1+e^{-\eta },\;\;g_{0}\left(\eta \right)=e^{-\eta },\\ k_{0}\left(\eta \right)=\eta e^{-\eta },\;\;h_{0}\left(\eta \right)=e^{-\eta } \end{array}$$

satisfying the following properties:

(15) $$\begin{array}{c} {ℒ}_{1}\left[c_{1}+c_{2}e^{\eta }+c_{3}e^{-\eta }\right]=0,\;\;{ℒ}_{2}\left[c_{4}e^{\eta }+c_{5}e^{-\eta }\right]=0. \end{array}$$

### 3.1. Zeroth Order Deformation Problems

The problems at the zeroth order are given by

(16) (1−p)ℒ1[f~(η,p)−f0(η)]=pħN1[f~(η,p),g~(η,p)],(1−p)ℒ2[g~(η,p)−g0(η)]=pħN2[f~(η,p),g~(η,p)],(1−p)ℒ2[k~(η,p)−k0(η)]  =pħN3[f~(η,p),g~(η,p),k~(η,p),h~(η,p)],(1−p)ℒ2[h~(η,p)−h0(η)]  =pħN4[f~(η,p),g~(η,p),k~(η,p),h~(η,p)],

where

(17) N1[f~(η,p),g~(η,p)]  =∂3f~(η,p)∂η3−(∂f~(η,p)∂η)2+α2(g~(η,p))2   +2f~(η,p)∂2f~(η,p)∂η2−λ∂5f~(η,p)∂η5+1,N2[f~(η,p),g~(η,p)]  =∂2g~(η,p)∂η2−2g~(η,p)∂f~(η,p)∂η   +2f~(η,p)∂g~(η,p)∂η−λ∂4g~(η,p)∂η4,N3[f~(η,p),g~(η,p),k~(η,p),h~(η,p)]  =∂2k~(η,p)∂η2−k~(η,p)∂f~(η,p)∂η   +αh~(η,p)g~(η,p)+2f~(η,p)∂k~(η,p)∂η   −λ∂4k~(η,p)∂η4,N4[f~(η,p),g~(η,p),k~(η,p),h~(η,p)]  =∂2h~(η,p)∂η2−αg~(η,p)k~(η,p)   −h~(η,p)∂f~(η,p)∂η+2f~(η,p)∂h~(η,p)∂η   −λ∂4h~(η,p)∂η4,

in which *p* ∈ [0,1] is the embedding parameter and *ħ* is the auxiliary nonzero parameter. By Taylor's theorem,

(18) $$\begin{array}{c} \tilde{f}\left(\eta ,p\right)=f_{0}\left(\eta \right)+\sum_{m=1}^{\infty }f_{m}\left(\eta \right)p^{m},\\ \tilde{g}\left(\eta ,p\right)=g_{0}\left(\eta \right)+\sum_{m=1}^{\infty }g_{m}\left(\eta \right)p^{m},\\ \tilde{k}\left(\eta ,p\right)=k_{0}\left(\eta \right)+\sum_{m=1}^{\infty }k_{m}\left(\eta \right)p^{m},\\ \tilde{h}\left(\eta ,p\right)=h_{0}\left(\eta \right)+\sum_{m=1}^{\infty }h_{m}\left(\eta \right)p^{m}. \end{array}$$

### 3.2. mth Order Deformation Problems

The general HAM equation for mth order can be given by

(19) $$\begin{array}{c} {ℒ}_{1}\left[f_{m}\left(\eta \right)-\chi_{m}f_{m-1}\left(\eta \right)\right]=\hbar R_{1,m}\left(\eta \right),\\ {ℒ}_{2}\left[g_{m}\left(\eta \right)-\chi_{m}g_{m-1}\left(\eta \right)\right]=\hbar R_{2}\left(\eta \right),\\ {ℒ}_{2}\left[k_{m}\left(\eta \right)-\chi_{m}k_{m-1}\left(\eta \right)\right]=\hbar R_{3,m}\left(\eta \right),\\ {ℒ}_{2}\left[h_{m}\left(\eta \right)-\chi_{m}h_{m-1}\left(\eta \right)\right]=\hbar R_{4,m}\left(\eta \right) \end{array}$$

with the following boundary conditions:

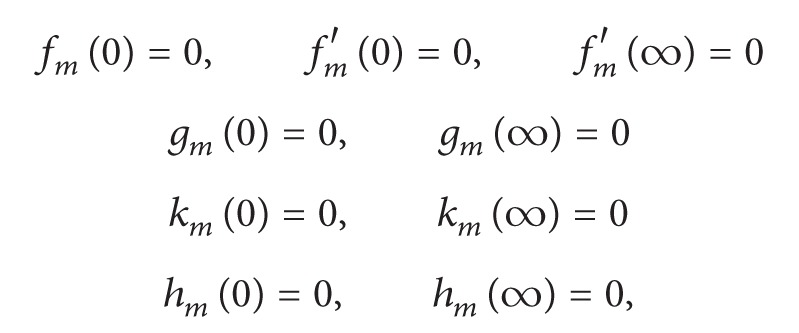
(20)

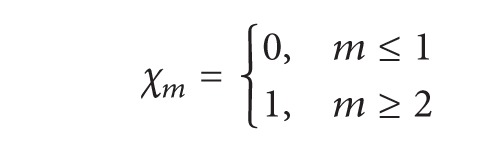
(21)

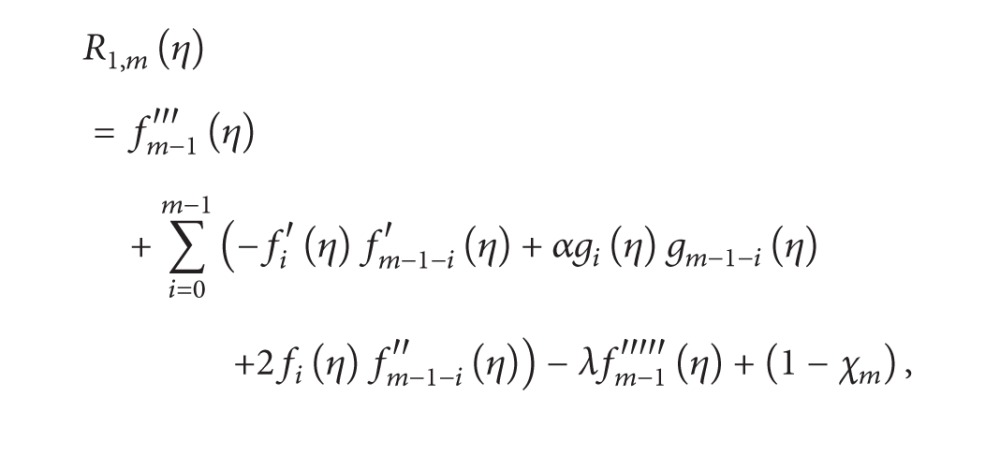
(22)

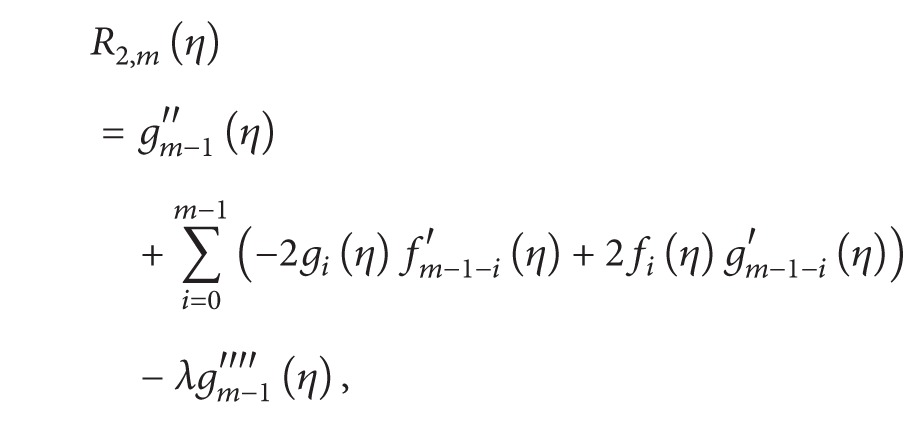
(23)

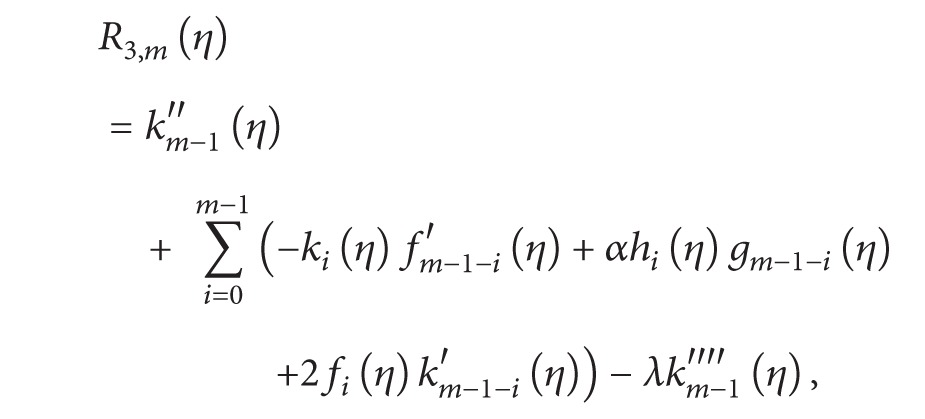
(24)

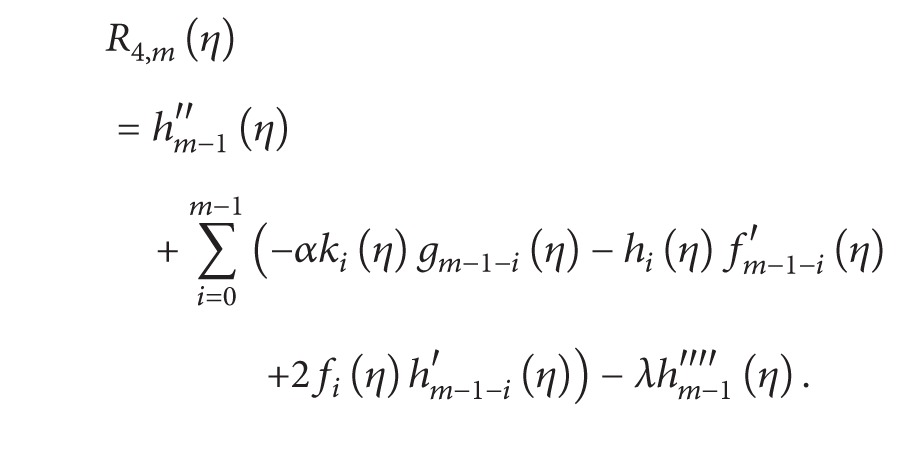
(25)
According to the above-defined method, the linear equations (19) with the boundary conditions (20) in the order *m* = 1,2, 3,… can be solved easily by means of any computational software, such as Mathematica/MATLAB.

## 4. Results and Discussion

In this paper, the effect of couple stress fluid on steady stagnation point flow over a rotating disk has been examined. The system of nonlinear ordinary differential equations (7) under the boundary conditions (8) has been solved analytically by employing HAM. The physical aspects of the considered problem have been examined for the radial velocity *f*′(*η*), azimuthal velocity *g*(*η*), and induced velocity functions *k*(*η*) and *h*(*η*) that arise due to the nonalignment in the flow axis and rotating disk axis in account of rotational parameter *α* and couple stress parameter *λ*.

**Figure 2:**
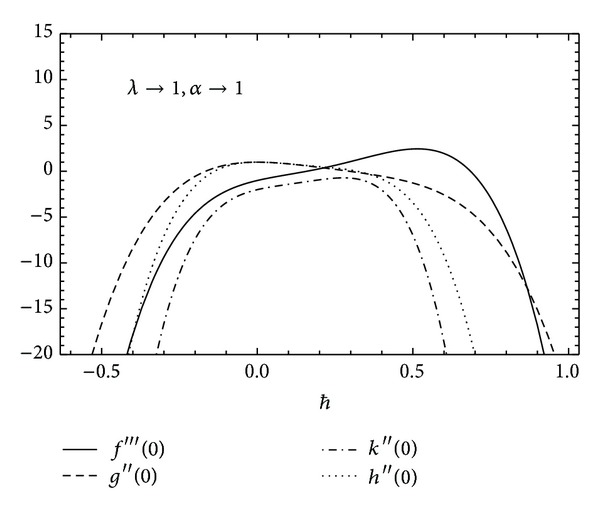
The *ħ* curves for *f*′′′(0), *g*′′(0), *k*′′(0), and *h*′′(0).

**Figure 3:**
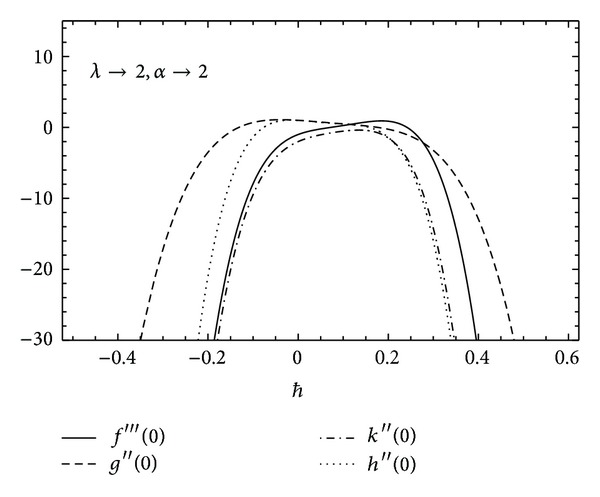
The *ħ* curves for *f*′′′(0), *g*′′(0), *k*′′(0), and *h*′′(0).

To ensure the convergence of HAM for the obtained solutions, it is necessary to find the appropriate value of *ħ* for which the results converge to the exact solution. In Figures 2 and 3 the *ħ* graphs are computed for*f*′′′(0), *g*′′(0), *k*′′(0), and *h*′′(0) to achieve the convergence region and the admissible range of values for different values of rotational and couple stress parameters.

**Figure 4:**
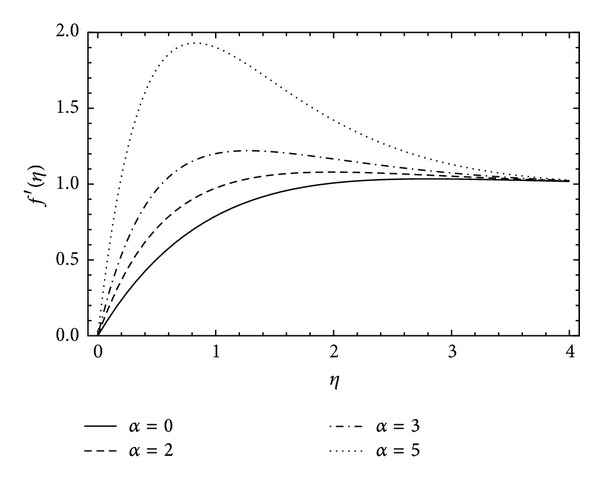
The influence of the rotation parameter *α* on *f*′(*η*) for *λ* = 1, *ħ* = −0.1.

**Figure 5:**
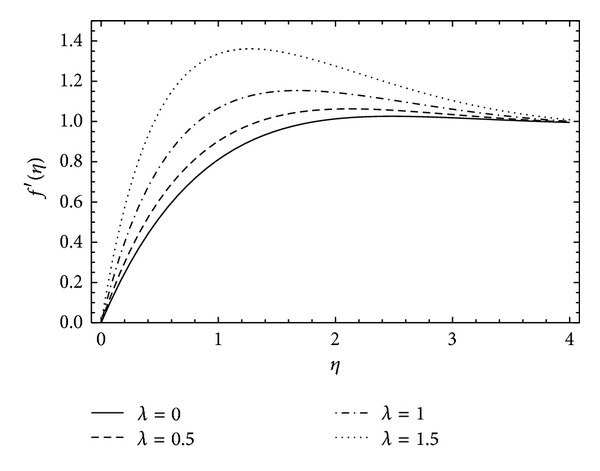
The influence of the couple stress parameter *λ* on *f*′(*η*) for *α* = 1, *ħ* = −0.2, and *λ* = 0 (Newtonian case).

Figure 4 illustrated the profile of radial velocity *f*′(*η*) against the rotational parameter *α*. It can be seen that there is an increase in the radial velocity with the increase in *α*, and also a rapid increment in velocity has been observed with high rotational parameter but as the flow approaches infinity it all converges at unity. The effect of couple stress parameter *λ* on the radial velocity *f*′(*η*) has been noticed in Figure 5. With the rotation in the disk and increasing couple stresses the flow velocity also increases. For *λ* = 0 the flow retains the Newtonian fluid.

**Figure 6:**
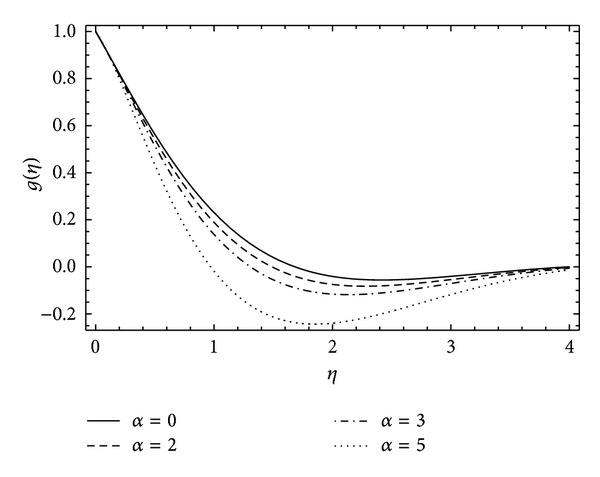
The influence of the rotation parameter *α* on *g*(*η*) for *λ* = 1, *ħ* = −0.2.

**Figure 7:**
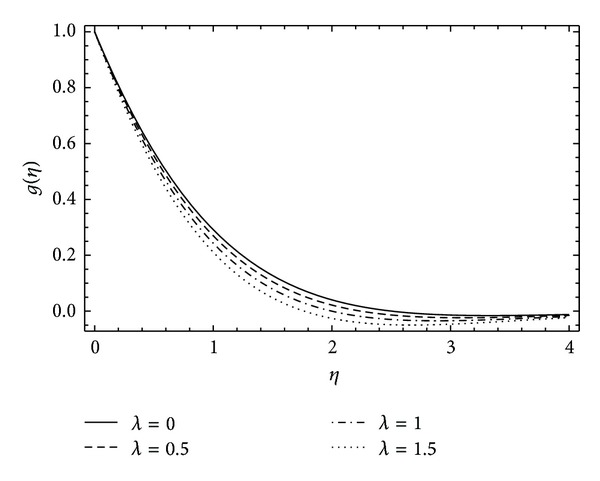
The influence of the couple stress parameter *λ* on *g*(*η*) for *α* = 2, *ħ* = −0.1, and *λ* = 0 (Newtonian case).

The insight of azimuthal velocity *g*(*η*) can be perceived in Figures 6 and 7. The influence of increasing rotation in the disk results in the decrease in flow velocity in azimuthal direction (see Figure 6). Also increase in the magnitude of couple stress parameter *λ* causes reduction in the azimuthal velocity *g*(*η*) as shown in Figure 7.

**Figure 8:**
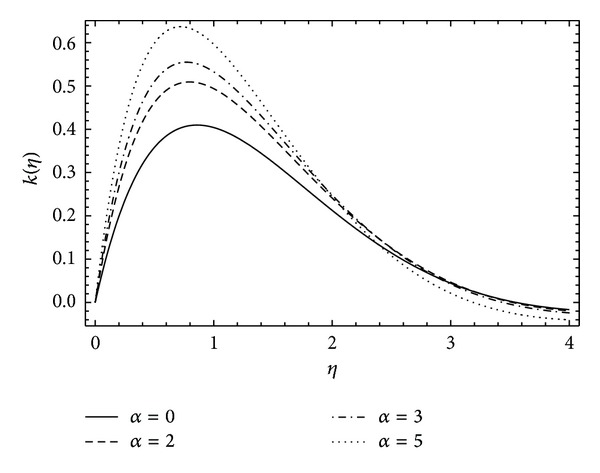
The influence of the rotation parameter *α* on *k*(*η*) for *λ* = 1, *ħ* = −0.1.

**Figure 9:**
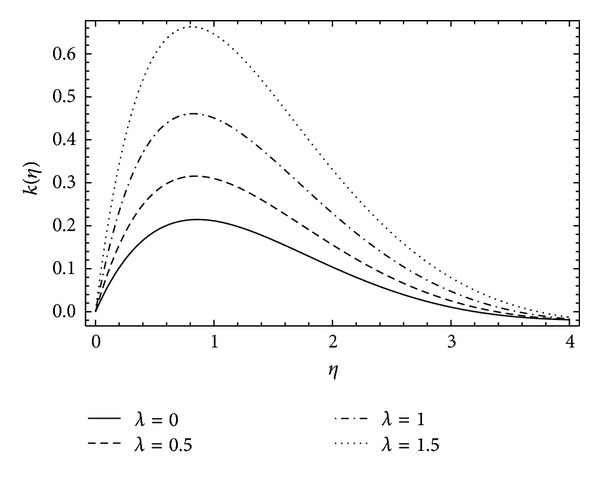
The influence of the couple stress parameter *λ* on *k*(*η*) for *α* = 1, *ħ* = −0.1, and *λ* = 0 (Newtonian case).

**Figure 10:**
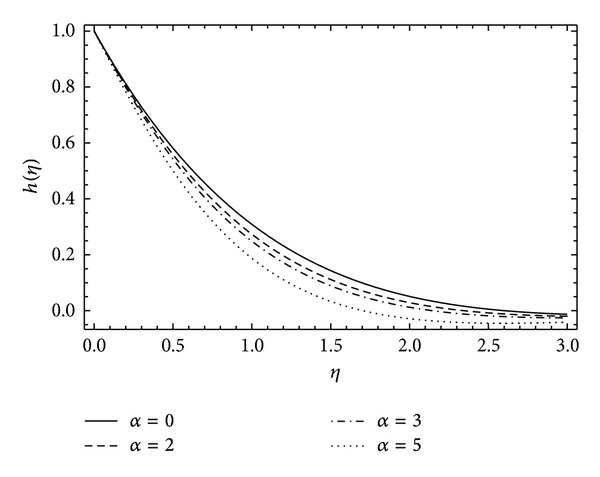
The influence of the rotation parameter *α* on *h*(*η*) for *λ* = 1, *ħ* = −0.1.

**Figure 11:**
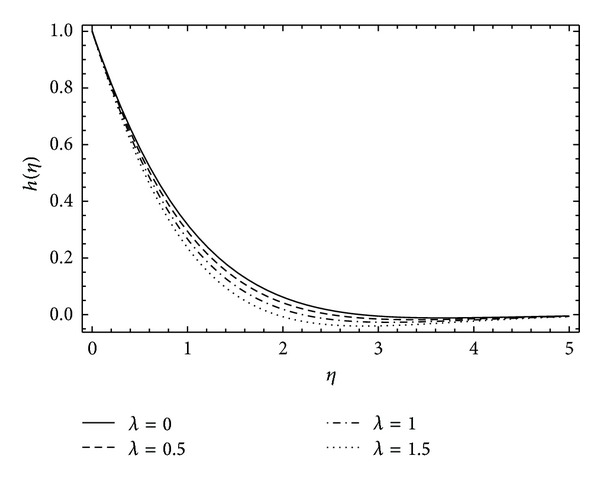
The influence of the couple stress parameter *λ* on *h*(*η*) for *α* = 1, *ħ* = −0.1, and *λ* = 0 (Newtonian case).

In Figure 8, the induced velocity function *k*(*η*) shows the rapid rise and then exponential decay in the fluid flow as the rotational parameter *α* increases. A similar behavior is observed in Figure 9 for velocity function *k*(*η*) with the varying values of couple stress parameter *λ*. It can be depicted in Figure 10 that the induced velocity function *h*(*η*) falls, decreasing the boundary wall thickness with the increase in rotational parameter *α*.  Figure 11 demonstrates the induced velocity profile of *h*(*η*) for different values of *λ*. It reveals that the presence of stresses in the fluid does not support the off-centered velocity function.

## 5. Conclusion

The present paper modelled the nonaligned flow of a couple stress fluid towards a rotating disk. The governing equations transformed to ordinary differential equations with the help of suitable similarity transformation. The analytical computation has been carried out by the homotopy analysis method and the influences of the pertinent parameters are investigated on the velocity profiles of the fluid in radial, azimuthal, and off-centered directions. Present investigation reveals that the couple stress parameter *λ* and disk rotational parameter *α* have the same effect of increasing radial velocity and decreasing the remaining velocity functions with the increase in respective parameters. It can be concluded from the above study that the rotation in the fluid particles and rotation in the disk give the combined effect of rotation in the fluid. Also, the equations for the shear stress and torque experienced by the disk have been evaluated for the couple stress flow and it was found that the torque remains unchanged with the off-centered flow.

## Notations

(*x*, *y*, *z*): Space coordinates
(*u*, *v*, *w*): Velocity components
*Ω*: Angular velocity
*ρ*: Density
*μ*: Viscosity
*ν*: Kinematic viscosity
*a*: Strength of flow
*γ*: Couple stress parameter
*b*: Distance between disk axis and flow axis
*α*: Nondimensional rotational parameter
*λ*: Dimensionless couple stress parameter
(*r*, *θ*): Cylindrical coordinates
*p*: Pressure of the fluid
*R*: Radius of the disk
*M*: Torque experienced by disk
*τ*_*x*_, *τ*_*y*_: Components of shear stress.
